# Controlled Synthesis of Atomically Layered Hexagonal Boron Nitride via Chemical Vapor Deposition

**DOI:** 10.3390/molecules21121636

**Published:** 2016-11-29

**Authors:** Juanjuan Liu, R. Govindan Kutty, Zheng Liu

**Affiliations:** 1 School of Geography and Environmental Sciences, Guizhou Normal University, Guizhou 550001, China; liujuanj@hotmail.com; 2 Center for Programmable Materials, School of Material Science and Engineering, Nanyang Technological University, Singapore 637798, Singapore; govindankuttyr90@gmail.com

**Keywords:** chemical vapor deposition, hexagonal boron nitride, synthesis, 61.72.jd, 61.72.uj, 68.37.Og

## Abstract

Hexagonal boron nitrite (h-BN) is an attractive material for many applications including electronics as a complement to graphene, anti-oxidation coatings, light emitters, etc. However, the synthesis of high-quality h-BN is still a great challenge. In this work, via controlled chemical vapor deposition, we demonstrate the synthesis of h-BN films with a controlled thickness down to atomic layers. The quality of as-grown h-BN is confirmed by complementary characterizations including high-resolution transition electron microscopy, atomic force microscopy, Raman spectroscopy and X-ray photo-electron spectroscopy. This work will pave the way for production of large-scale and high-quality h-BN and its applications as well.

## 1. Introduction

Hexagonal boron nitride (h-BN), as a typical large-bandgap 2D crystal, has been attracting increasing attention due to its unique properties such as high thermal conductivity and excellent electrical insulation, etc. [1,2,3,4,5,6,7]. Due to its atomically smooth surface and close in-plane lattice match to graphene, h-BN has been considered an important layered material complementary to graphene [8,9,10,11,12]. Previous work reported that h-BN can help to improve the mobility of graphene as a substrate [5], fabricate high current cut-off devices for radio frequency applications [12], engineer a bandgap in stacked graphene/h-BN (G/h-BN) [10,13,14,15,16], and enhance the ON/OFF ratio of the graphene field effect transistor (GFET) in the vertical G/h-BN heterostructure [11]. These progresses, together with the high thermal conductivity, thermal stability and large bandgap, make the h-BN 2D crystal a promising component in both fundamental uses and applications such as optics, electronics, etc.

Unlike the mature technology of the production of highly-oriented pyrolytic graphite (HOPG), the synthesis of bulk h-BN crystals such as highly-oriented pyrolytic boron nitride (HOPBN) [17] is still a great challenge, although a few works have reported on the synthesis of h-BN crystals using high-temperature and high-pressure methods [18,19,20]. There is an urgent need to develop an alternative approach to prepare large-scale and high-quality h-BN for further applications for next-generation electronics. Previous efforts have demonstrated that atomically layered h-BN can grow on copper foil, using the chemical vapor deposition (CVD) method, with ammonia borane as a precursor at a temperature from 900 °C to 1000 °C [2,21] Some further works, with optimized growing conditions, have advanced the thicknesses of h-BN to a single layer [22,23]. In most of the recent works, h-BN films are grown on copper foil. The quality of such CVD h-BN cannot be compared with the exfoliated samples, and tends to a relatively low performance.

## 2. Results

**Figure 1:**
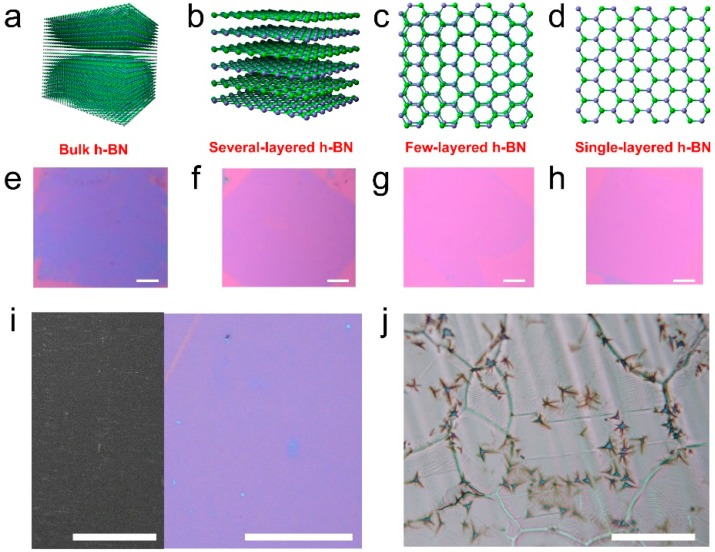
Optical images (**a**–**d**) and illustrations (**e**–**h**) of controllable growth of h-BN on Ni substrates for bulk (**a**,**e**); several layers (**b**,**f**); few layers (**c**,**g**) and a single layer (**d**,**h**) on SiO_2_. Scale bar in (**e**–**h**) is 2 mm; (**i**) SEM image of few-layered h-BN on Ni foil (left, scale bar 1 mm) and optical image of few-layered h-BN film on SiO_2_ (scale bar 100 µm); (**j**) Bulk h-BN domains on Ni foils. Scale bar: 100 µm.

In this paper, we demonstrated that, with Ni as a substrate and at a high temperature (1000~1200 °C), high-quality h-BN can be prepared. The thicknesses of as-grown h-BN can be well controlled, from single-layered, or few-layered, to even bulk, as the optical images show in Figure 1.

Figure 1 shows optical images and illustrations of as-transferred h-BN films. Figure 1a–d are illustrations of bulk, six-layer, double-layer and single-layer h-BN, respectively. Figure 1e–h are corresponding optical images of as-transferred h-BN films on SiO_2_ substrates (thickness: 285 nm). Their layer numbers from left to right are >10, five to six, two to three, and one to two layers, respectively. The thick h-BN on SiO_2_ is green while thinner h-BN turns to light purple. We noticed that, for several-layer h-BN (>3 nm), the thickness uniformity and coverage is not as good as for few-layered h-BN on Ni (Supplementary Materials Figure S1). As it can be seen in Figure 1e, there are tiny holes in the film. The h-BN films in Figure 1e–h were prepared at a growing temperature of 1000 °C (Figure 1i). If the growing temperature is high (>1100 °C) and the duration is long (>1 h), high crystalline h-BN stars can be found (Figure 1j).

The thicknesses of CVD h-BN can be well controlled from a single layer to bulk with optimized growing conditions. We should note that the temperature is critical to control the thickness of h-BN. A high temperature up to 1100 °C is required in order to synthesize thick h-BN film. The melting point of Cu foil is around 1050 °C while Ni foils resist higher temperatures up to 1400 °C. So, the substrate (Ni foil) is important to produce h-BN films with a controlled thickness. The growing conditions are listed in Table 1. Monolayer h-BN can be synthesized at under ~1000 °C by heating a precursor as low as 40 °C. The growing time is 1 h. A longer growing time results in a thicker h-BN film. As expected, a higher precursor temperature and growing temperature will enhance the growing rate of h-BN. Figure 1i is a close-up area of few-layered h-BN film. A high furnace temperature (1200 °C) and precursor temperature will result in very thick h-BN up to tens of nanometers, as shown in Figure 1j. The h-BN flakes can be observed in the optical images. Each flake has a few legs and the angles between them are multiples of 30°. We also realized that the domain orientation may play an important role in the formation of h-BN, especially at high temperatures. At low temperatures, the ammonia borane precursors are not fully condensed into h-BN, and therefore it results in relatively low-quality but high-coverage h-BN films (Figure S1). At high temperatures, highly crystalline h-BN can be easily obtained but is also highly selective to the lattice orientation of the domains of the substrates. Therefore, we can only observe thick h-BN flakes of various thicknesses at Ni foils at high temperatures (Figure S2). This is also confirmed by previous reports [24].

**Figure 2:**
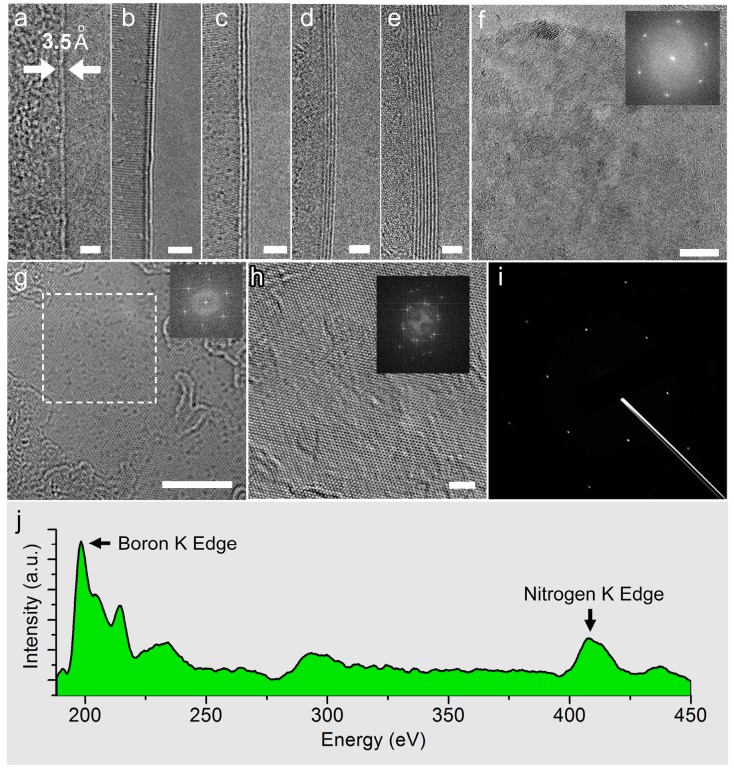
TEM images, diffractions and EELS of single-layered and few-layered h-BN films. (**a**–**e**) TEM images of single- (1 L), double- (2 L), three- (3 L), four- (4 L) and seven-layered (7 L) h-BN films, respectively. Scale bar: 2 nm; (**f**) TEM image of random area of single-layered film and its FFT pattern (inset). Scale bar: 10 nm; (**g**) HRTEM image of single-layered h-BN film and its FFT patterns from the white-dashed region. Scale bar: 5 nm; (**h**) TEM image of few-layered h-BN and its FFT patterns. Scale bar: 2 nm; (**i**) A typical diffraction pattern of few-layered h-BN in a large area; (**j**) EELS of few-layered h-BN film.

Figure 2 shows the TEM images, diffraction patterns and elemental analysis of single-layered and few-layered h-BN films. Figure 2a–e show the edges of single-layered (1 L), double-layered (2 L), three-layered (3 L), four-layered (4 L) and seven-layered (7 L) h-BN films, respectively. The scale bar is 2 nm. The thickness of an individual h-BN layer is estimated to be ~0.35 nm. Figure 2f shows the TEM image of single-layered h-BN film. The scale bar is 10 nm. The inset is the corresponding fast Fourier transform (FFT) pattern, showing individual six-fold symmetry spots. Figure 2g is a high-resolution TEM (HRTEM) image of single-layered h-BN film. The scale bar is 5 nm. The inset shows the corresponding FFT patterns. Figure 2h shows the morphology of few-layered h-BN and its FFT pattern (inset). Figure 2i is a typical diffraction pattern of a random area of few-layered h-BN film. The electron energy loss spectrum (EELS) is shown in Figure 2j. Both the boron edge (~188 eV) and nitrogen edge (~410 eV) are marked by black arrows.

## 3. Discussion

**Figure 3:**
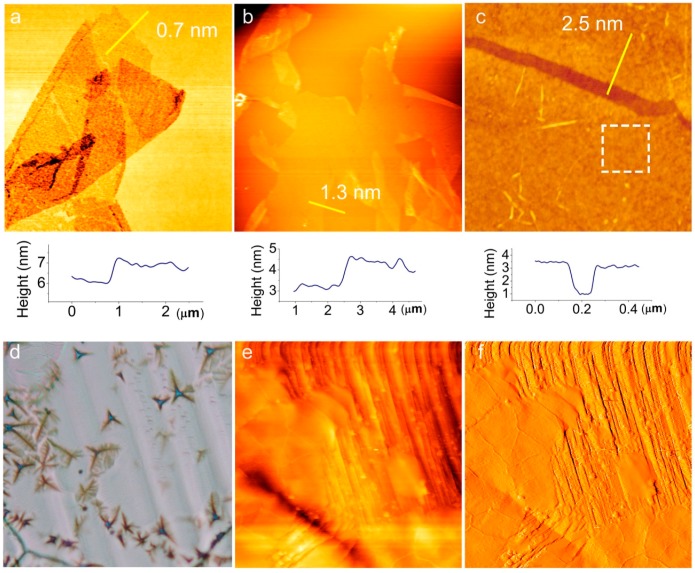
AFM morphologies of as-transferred h-BN on SiO_2_ with thicknesses of 0.78 nm (**a**); 1.3 nm (**b**) and 2.5 nm (**c**), respectively, corresponding to one- to two-layered, three- to four-layered and six- to seven-layered h-BN. Inset of each image shows the height profile of the cross-sections. The sizes of Figure (**a**–**c**) are 10 µm × 10 µm, 30 µm × 30 µm= and 1.5 µm × 1.5 µm; (**d**) Optical image of h-BN bulk; (**e**,**f**) Topography and corresponding current sensing AFM image of h-BN on Ni foil. Size: 20 µm × 20 µm. Thickness: ~20 nm.

The thicknesses and morphologies of h-BN films are further confirmed by AFM in Figure 3. Figure 3a–c show the height topography of atomically layered h-BN on SiO_2_. Their sizes are 10 µm × 10 µm, 30 µm × 30 µm and 1.5 µm × 1.5 µm, and the typical thicknesses are 0.7, 1.3 and 2.0 nm (corresponding to 1~2 L, 3~4 L, 5~6 L h-BN), respectively, considering the substrate effect. The roughness of the thin layer h-BN can be as low as ~300 pm (the dashed region in Figure 3c), comparable to the value of SiO_2_ (~250 pm) [12]. Figure 3d shows thick h-BN domains on a Ni foil. Figure 3e,f are the height topography and corresponding current sensing image of the h-BN thick film. The thickness is ~20 nm (more than 50 layers). Due to the insulating behavior of h-BN, the h-BN films can be easily found from the current sensing image.

**Figure 4:**
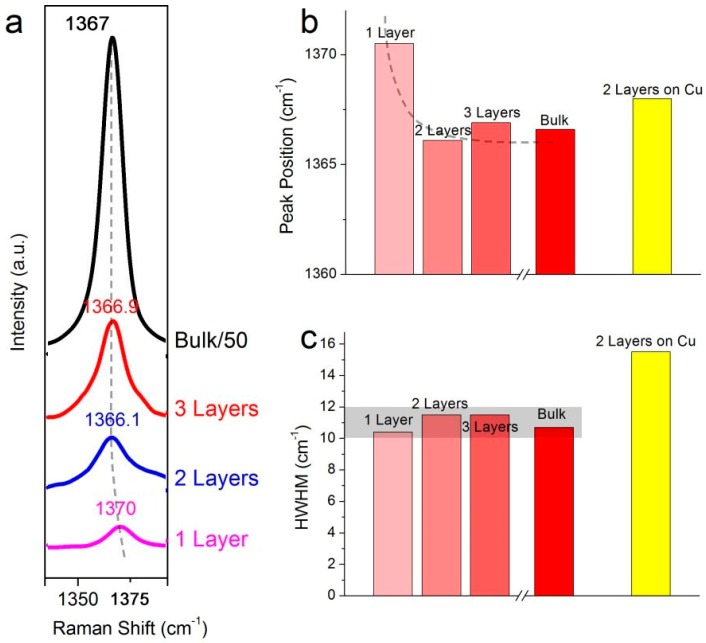
Raman signature of h-BN films. (**a**) Raman spectra of one-layer, two-layer and three-layer h-BN films and bulk h-BN; (**b**) Peak position (E_2g_) of h-BN film versus thickness; (**c**) HWHM of E_2g_ peak versus thickness.

Raman spectroscopy is a powerful technique to determine the thickness and quality of as-grown h-BN films. Figure 4a shows typical Raman spectra taken from one-layer, two-layer, three-layers and bulk h-BN film with a 514.5 nm laser excitation which was performed at a power of 20 mW. The Raman vibrating mode of h-BN (E_2g_) locates at around 1368 cm^−1^ [25,26]. From bulk to single-layer h-BN films, the E_2g_ peak shifts from 1367 to 1371 cm^−1^, along with a dramatic drop of the Raman intensity (Figure 4a,b). For most single-layered h-BN on SiO_2_, these results agree with a previous report on the Raman signature of mechanical exfoliated h-BN films [7]. Also, the half width at half-maximum (HWHM) of E_2g_ peaks is analyzed to evaluate the quality of h-BN films (Figure 4c). For most h-BN samples grown on Ni foil, the HWHM are ~11 cm^−1^, comparable to the value of mechanical exfoliated h-BN (10~12 cm^−1^). As a comparison, the HWHM of E_2g_ peak from h-BN on a Cu substrate is ~16 cm^−1^. All these Raman data demonstrate that the high-quality and atomically layered h-BN films can be grown on Ni foil.

**Figure 5:**
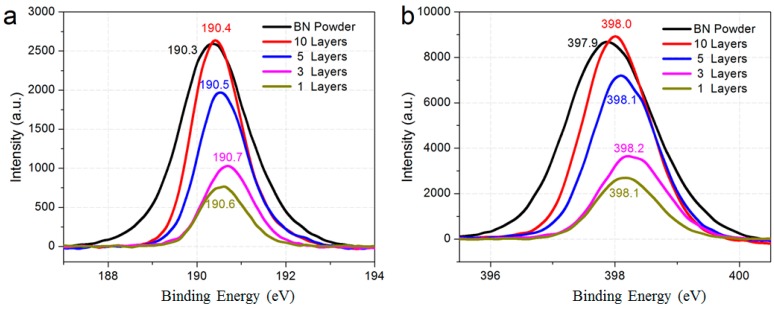
XPS signature of h-BN films. (**a**,**b**) XPS spectra of B and N 1s core level of samples in various thicknesses.

The XPS spectra of h-BN films with various thicknesses are represented in Figure 5a,b. It was previously reported that the B 1s core level of hexagonal boron nitride locates at ~190 eV and the N 1s core level locates at 398 eV. For the single-layered and few-layered h-BN film that was grown, the peak from B located from 190.3 to 190.7 eV, and the peak from N locate from 379.9 to 398.2 eV, consistent with the reported values. Furthermore, h-BN powder is used for comparison. The B and N peak positions are ~190.3 and 397.9 eV, close to the value from CVD growth. The atomic concentration of the h-BN that was grown is 50% ± 4%, showing an excellent stoichiometry ratio of B and N. The slight redundancy may come from the nitric residual during transfer. Also, under the same conditions, the intensity of h-BN becomes weak while the h-BN film is less than five layers.

**Figure 6:**
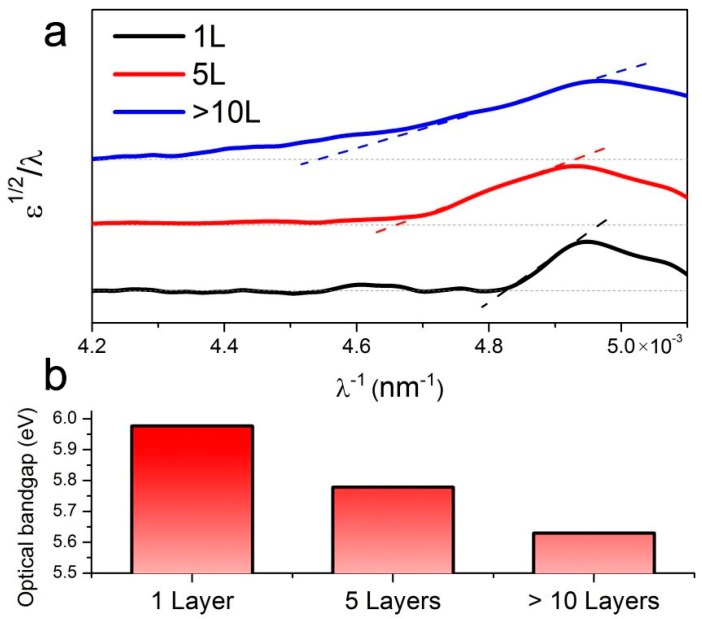
Ultraviolet-visible adsorption spectra of h-BN films of various thicknesses taken at room temperature. (**a**) UV adsorption spectra of 1 L, 5 L and thick (>10 L) h-BN films (ε^1/2^/λ versus 1/λ); (**b**) Calculated optical bandgap for each h-BN film.

To find out how the thickness depends on the thicknesses of h-BN, we further collected the UV-visible absorption spectrum of the atomically layered h-BN film (Figure 6a). The h-BN samples (~1 cm × 1 cm) were first transferred to quartz substrates. Before measurements, a blank quartz plate was used to collect the background which was subtracted in the following measurements of as-transferred h-BN films. The bandgap of h-BN films, *E_g_*, was derived from Tauc’s equation ω^2^ε = (*h*ω − *E_g_*)^2^, where ε is the optical absorbance and ω is the angular frequency of the incident radiation (ω = 2π/λ, and λ is the wavelength) [27,28,29] The optical bandgap *E_g_* = *hc/*λ*_g_* can be calculated from the intersection point of *ε*^1/2^/λ with the x-axis. Such estimation requires the plot of *ε*^1/2^/λ versus 1/λ to be a straight line in the corresponding spectral range, which is clearly shown in Figure 6b. The optical bandgap for thick h-BN is ~5.6 eV (220 nm), close to the previous experimental report on h-BN bulk [27,28,29] and the calculations [30,31]. For the few-layered h-BN, the optical bandgap is ~5.8 eV, consistent with our previous study on the few-layered h-BN grown on Cu substrates [32]. The maximum optical bandgap is found in single-layered h-BN with a value of ~6.0 eV, as shown in Figure 5b, which excellently meets the theoretically predicated value [15]. 

## 4. Materials and Methods

The growth of h-BN was carried out in a quartz tube furnace. A nickel foil with a thickness of 25 μm (Purity: 99.9%, American Elements, Inc., 1093 Broxton Ave. Suite 2000, Los Angeles, CA, USA) was put in the center of the quartz tube furnace. The quartz tube was pump down to 60 mT during growth. The temperature was raised to 1000 °C in 50 min under the protection of 300 mT Ar/H_2_ gas flow (15 vol % H_2_ balanced by 85 vol % Ar), and subsequently ammonia borane (NH_3_-BH_3_), as precursors, was sublimated at ~40–80 °C as the source of B and N. The typical growth time is about 1–2 h. The temperature of ammonia borane and the growing time are two main factors to control the thickness of h-BN film. After growth, the furnace cooled down to room temperature slowly. Therefore, the Ni foils were coated with poly(methyl methacrylate) (PMMA) to transfer h-BN to other substrates for further characterizations. High resolution transmission electron microscopy (HRTEM, JEOL-2100) and elemental mapping (Gatan GIF) were employed for selected area electron diffraction (SAED), electron energy loss spectroscopy (EELS) measurements and atomic image. Atomic force microscopy (AFM, Agilent PicoScan 5500 and Veeco Digital Instrument Nanoscope IIIA was used to obtain thicknesses and topographical variations of the samples. X-ray photoelectron spectroscopy (XPS) was performed using monochromatic aluminum KR X-rays. Raman spectroscopy (Renishaw inVia, was performed at 514.5 nm laser excitation. 

## 5. Conclusions

In this work, we show that by controlling the growing temperature and reaction time, we can produce highly crystalline h-BN film from star-like shapes down to a few layers. The high-quality as-grown h-BN is confirmed by our characterization. The h-BN peaks can be in the Raman spectra and HRTEM shows the clean surface of the h-BN film, as well as the hexagonal lattice. XPS confirms a 1:1 ratio of the B and N elements in the sample. Also, the absorption spectra indicate the bandgap evolution of h-BN from 5.8 eV (bulk) to 6.0 eV (a few layers). Our work will shed light on the large-scale production of high-quality ultra-thin h-BN films for their applications including heat dissipation and anti-oxidation coating.

## Figures and Tables

**Table 1:** Specific growth conditions of h-BN films in various layers.

Thickness of h-BN	1–2 L	2–3 L	5–6 L	Few Layers	Bulk
Growth temperature	1000 °C	1000 °C	1000 °C	1000 °C	>1100 °C
Growth time	1 h	1.5 h	1 h	1 h	0.5 h
Precursor temperature	40 °C	40 °C	50 °C	70 °C	70 °C

## Supplementary Materials

Supplementary materials can be accessed at: http://www.mdpi.com/1420-3049/21/12/1636/s1.
