# Supplementary material for: Controlled Synthesis of Atomically Layered Hexagonal Boron Nitride via Chemical Vapor Deposition
Source: Molecules. 2016 Nov 29;21(12):1636. doi: 10.3390/molecules21121636 (PMC6274273; doi:10.3390/molecules21121636)
Supplement: Supplementary file 1 [file molecules-21-01636-s001.pdf]

Supplementary Materials: Controlled Synthesis of Atomically Layered Hexagonal Boron Nitride via Chemical Vapor Deposition

Juanjuan Liu, R. Govindan Kutty and Zheng Liu

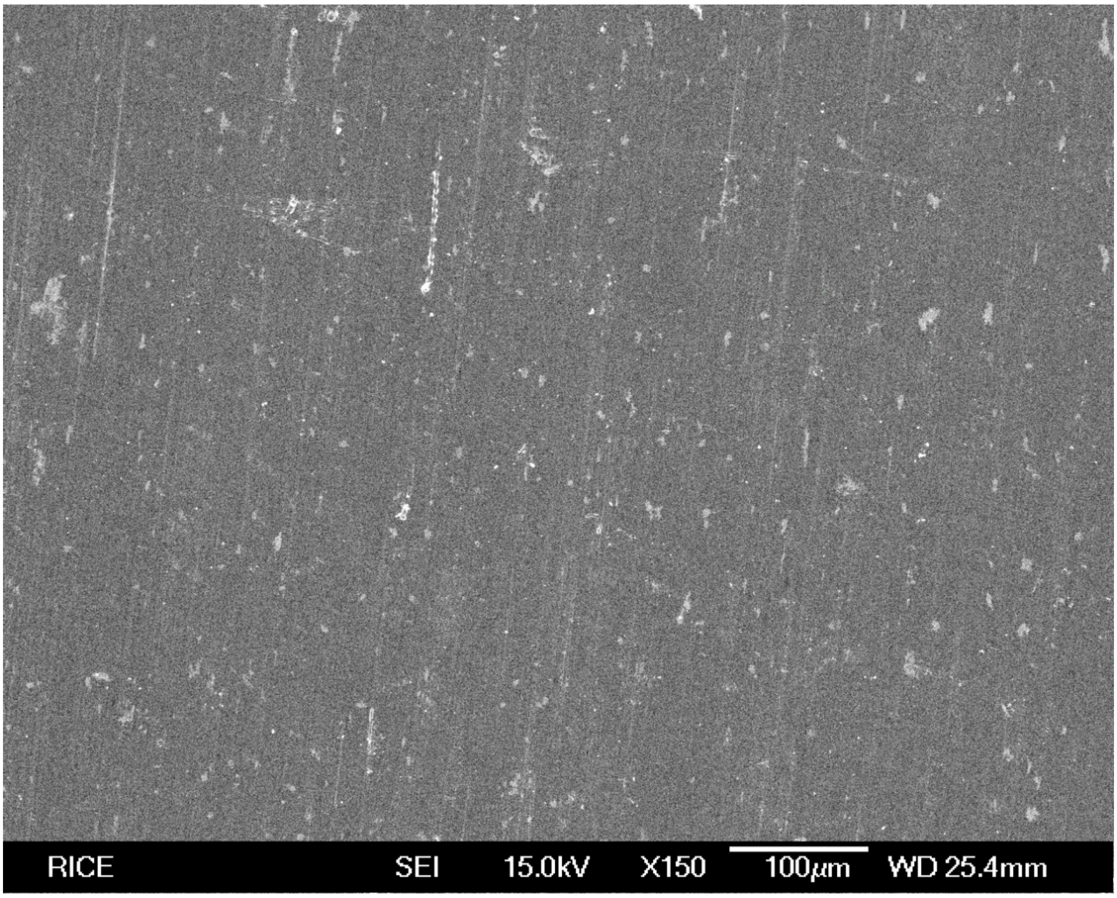

Figure S1. SEM image of high-coverage few-layered h-BN grown on N foils at low. The white areas are Ni while dark grey areas are h-BN due to their great different in electrical conductivity.

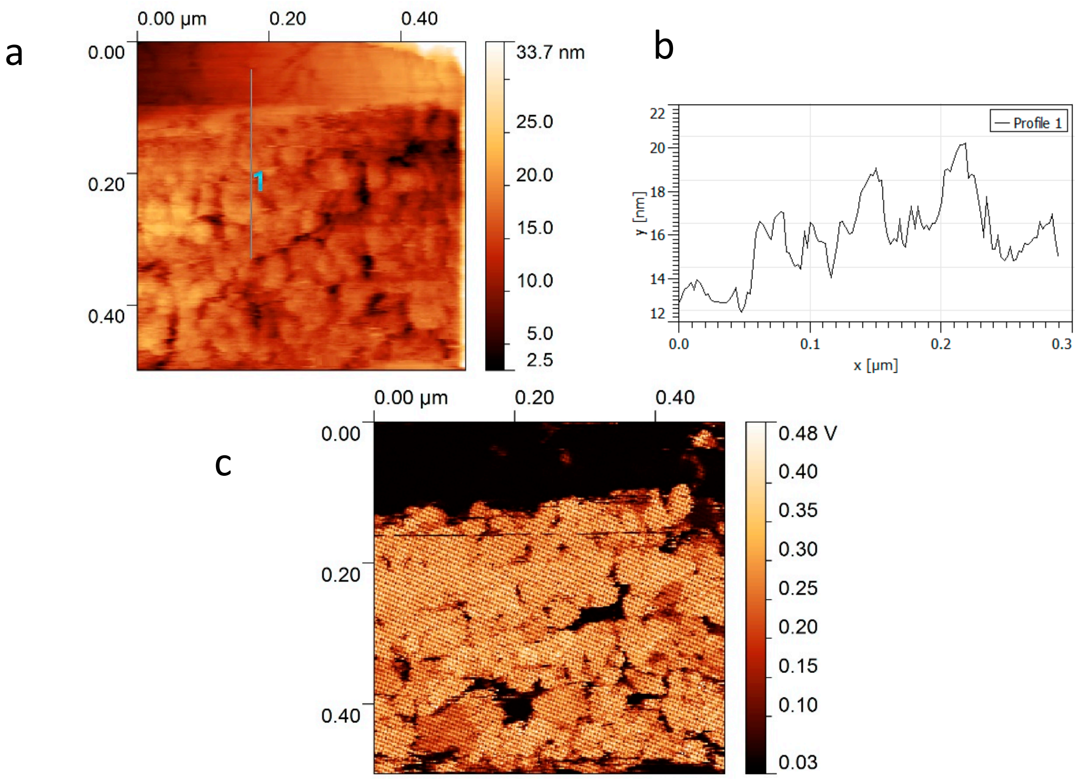

Figure S2. AFM image of low-coverage thick h-BN grown on Ni at high temperature. (**a**) Height topography of the h-BN fakes; (**b**) Height cross-section showing the thickness of h-BN is from 4–6 nm; (**c**) Electrical conductivity map of the h-BN. The yellow areas are h-BN and dark areas are Ni. The scanning size is 0.5 × 0.5 micron.
